# Tumor Necrosis Factor-Like Weak Inducer of Apoptosis (TWEAK)/Fibroblast Growth Factor-Inducible 14 (Fn14) Axis in Cardiovascular Diseases: Progress and Challenges

**DOI:** 10.3390/cells9020405

**Published:** 2020-02-11

**Authors:** Nerea Méndez-Barbero, Carmen Gutiérrez-Muñoz, Rafael Blázquez-Serra, Jose L. Martín-Ventura, Luis M. Blanco-Colio

**Affiliations:** Vascular Research Lab, IIS-Fundación Jiménez Díaz University Hospital, Av. Reyes Católicos 2, 28040 Madrid, Spain; carmen.gutierrezm@quironsalud.es (C.G.-M.); jlmartin@fjd.es (J.L.M.-V.)

**Keywords:** TWEAK, Fn14, vascular remodeling, stroke, heart failure

## Abstract

Cardiovascular diseases (CVD) are the leading cause of mortality in Western countries. CVD include several pathologies, such as coronary artery disease, stroke, peripheral artery disease, and aortic aneurysm, among others. All of them are characterized by a pathological vascular remodeling in which inflammation plays a key role. Interaction between different members of the tumor necrosis factor superfamily and their cognate receptors induce several biological actions that may participate in CVD. The cytokine tumor necrosis factor-like weak inducer of apoptosis (TWEAK) and its functional receptor, fibroblast growth factor-inducible 14 (Fn14), are abundantly expressed during pathological cardiovascular remodeling. The TWEAK/Fn14 axis controls a variety of cellular functions, such as proliferation, differentiation, and apoptosis, and has several biological functions, such as inflammation and fibrosis that are linked to CVD. It has been demonstrated that persistent TWEAK/Fn14 activation is involved in both vessel and heart remodeling associated with acute and chronic CVD. In this review, we summarized the role of the TWEAK/Fn14 axis during pathological cardiovascular remodeling, highlighting the cellular components and the signaling pathways that are involved in these processes.

## 1. Introduction

Cardiovascular diseases (CVD) are the main cause of death in developed countries, despite the fact that CVD rates and case-fatality rates have fallen considerably over the last two decades in those countries. CVD accounts for 17.3 million deaths per year, a number that is expected to grow to more than 23.6 million by 2030 [1]. These data indicate that CVD claims more lives than all forms of cancer combined. Direct and indirect costs of CVD and stroke total more than $320 billion, including health expenditures and lost productivity. CVD is a general term to define a group of heart and blood vessel disorders that include coronary heart disease, cerebrovascular disease, peripheral arterial disease, and aortic disease. Individuals at risk of CVD may show elevated blood pressure, glucose, and lipids, as well as be overweight or obese. The pathophysiological feature of cardiovascular disease is based on vascular/cardiac remodeling, which is the result of myocardium and vascular response to a range of hemodynamic, metabolic, and inflammatory stimuli. Remodeling is initially adaptive and functional, and comprises molecular, cellular, and interstitial changes that, when are sustained a long time, could cause clinical manifestations as pathological changes in size, shape, and function of the heart and vessels [2]. Remodeling involves responses not only of the cardiomyocytes, endothelial, and vascular smooth muscle cells (SMCs), but also of interstitial cells and matrix [3].

Several studies support the importance of the tumor necrosis factor (TNF) superfamily of proteins in cardiovascular remodeling. Ligands of the TNF superfamily are mainly expressed as type II transmembrane proteins. These ligands share a common structural motif called TNF homology domain (THD), which binds to cysteine-rich domains (CRDs) of TNF receptors [4]. Moreover, the ligands could be processed by proteases as soluble cytokines when their extracellular domain is cleaved. The TNF receptors superfamily are primarily type I transmembrane proteins, and could also be secreted as soluble proteins due to proteolytic processing, or an alternative splicing of the transmembrane domain encoding exon [5]. Tumor necrosis factor-like weak inducer of apoptosis (TWEAK) and its sole functional receptor fibroblast growth factor-inducible 14 (Fn14) are two members of the TNF superfamily that participate in multiple biological activities, including proliferation, migration, differentiation, apoptosis, angiogenesis, and inflammation [6]. All of these processes are closely related to pathological cardiovascular remodeling.

## 2. TWEAK and Fn14: Two Members Belonging to the TNF Superfamily

### 2.1. Structure of TWEAK and Fn14

**Figure 1:**
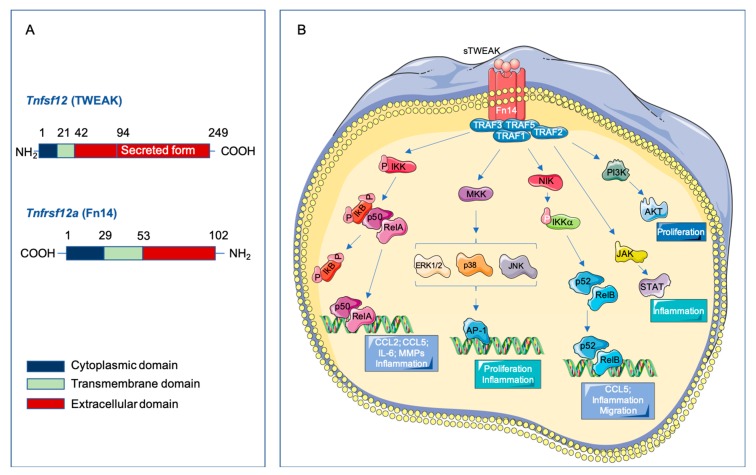
Tumor necrosis factor-like weak inducer of apoptosis (TWEAK)/ fibroblast growth factor-inducible 14 (Fn14) Signaling. (**A**) Schematic representation of human TWEAK and Fn14 receptor structure showing full-lengths and TWEAK secreted form. (**B**) Soluble TWEAK binds to the extracellular domain of the Fn14 receptor and produces its trimerization. This structural change of the receptor triggers the recruitment of TRAF proteins to its cytoplasmic tail and the activation of the different pathways; canonical (p50/RelA) and non-canonical (p52/RelB) NF-kB, ERK/JNK/p38 and AP-1, JAK/STAT and PI3K/AKT. Increased activation of these signaling pathways leads to the regulation of specific target genes with their biological mechanistic role.

The cytokine TWEAK (Apo3L, Tnfsf12) is a member of the tumor necrosis factor superfamily (TNFSF), described for the first time in 1997 [7]. The human TWEAK gene is located at chromosome position 17p13.1 and encodes a 249-amino acid (aa) type II transmembrane glycoprotein of 30 kDa (Figure 1A). The C-terminal extracellular domain contains the receptor binding site and, as other members of the TNF superfamily, has a consensus sequence motif for furin cleavage [8,9]. The N-terminal region contains a hydrophobic anchor, allowing its insertion into the cell membrane with the N-terminus inside the cell. Although TWEAK is initially synthesized as a membrane-bound protein (mTWEAK) that is processed quickly into a soluble fragment (sTWEAK), which mediates the different biological properties of this protein. The TWEAK receptor was erroneously identified in 1998 as the TNF receptor superfamily (TNFRSF) member death receptor 3 (DR3) [10]. In 2001, the TWEAK receptor was cloned and identified as the described human fibroblast growth factor-inducible 14 (Fn14, Tnfrsf12a) [11,12]. The human Tnfrsf12a gene is located at the chromosomal position 16p.13.3 and encodes a 129-aa type I transmembrane protein of 14 kDa (Figure 1A) [11]. Fn14 is processed into a 102-aa mature protein, being it the smallest TNFRSF member to date. The extracellular domain of Fn14 contains the TWEAK binding site [13]. The signaling induced by TNF superfamily receptors involves the presence of death domains in their cytoplasmic tail. However, the Fn14 cytoplasmic tail is too short to have a death domain, but it contains a TNF receptor-associated factor (TRAF)-binding site with three threonines that could be potentially phosphorylated to induce TRAF-binding and subsequent transmission of TWEAK signaling [14]. TWEAK trimerizes and binds to Fn14 monomers, promoting receptor trimerization and signal transduction [15]. Both, TWEAK and Fn14 are closely related in humans and mice, being that their homology is higher than 90%. In fact, murine TWEAK can bind to human Fn14 and vice versa [16]. This detail is of importance since it has recently been published that TWEAK does not cross-react with any other members of the TNF or TNFR superfamilies, being it’s interaction specific for Fn14 [16]. The phylogenetic conservation between different species suggests that the TWEAK/Fn14 axis plays an important biological role [17]. Finally, TWEAK- and Fn14-deficient mice are healthy and have a normal life span.

### 2.2. Expression of TWEAK and Fn14

TWEAK is ubiquitously expressed in many tissues, such as the heart, vasculature, pancreas, intestine, brain, lung, ovary, and skeletal muscle, and at low levels in the liver and kidney [7]. TWEAK is expressed in both healthy and pathological vessel walls [18]. By contrast, Fn14 expression is relatively low or absent in healthy tissue, including vasculature and heart, although it is highly and rapidly induced during pathological conditions [18]. Changes in Fn14 expression have been reported in experimental models of different pathologies, such as chronic liver injury [19], myocardial infarction [20], restenosis after balloon injury and femoral wire injury [11,21], atherosclerosis [18], acute kidney injury [22], or cardiac dysfunction [23], among others.

At the cellular level, TWEAK is expressed in various immune cell types, including macrophages [7], microglia [24], activated monocytes and T cells [25,26], dendritic cells, natural killer (NK) cells [27], and mast cells [28]. In addition, macrophages/monocytes are the main source of sTWEAK in inflammatory tissues [29]. Within the cardiovascular system, both TWEAK and Fn14 are expressed in cardiomyocytes [20,23,30,31], human endothelial cells [11] and SMCs [18]. Moreover, Fn14 mRNA is also detected in cardiac fibroblasts [32]. TWEAK protein secretion can be upregulated by PMA and INF-γ in cultured human peripheral mononuclear cells [27] and in natural killer cells [26] and by anti-DNP Immunoglobulin E (IgE) sensitization followed by DNP challenge in bone marrow–derived mast cells (BMMCs) [28]. In addition, Fn14 expression is upregulated under pathological conditions by several growth factors, cytokines and interleukins. Thus, Fn14 expression is increased by pro-inflammatory cytokines (IL-1B and INF-γ), growth factors (PDGF-BB, EGF, FGF-2), angiotensin II, or α-thrombin [11,18] in human and rat aortic SMCs. Moreover, Fn14 expression is upregulated in human umbilical endothelial cells by VEGF-A and FGF-2 [33], and in mouse aortic endothelial cells by histamine and the platelet-activator factor (PAF) [28]. In neonatal rat cardiomyocytes, Fn14 expression is induced by fibroblast growth factor 1 (FGF-1), norepinephrine, angiotensin II, and mechanical stretch via the Rho/ROCK pathway [30]. Furthermore, Fn14 expression is induced by fetal bovine serum, EGF, FGF-1, FGF-2, PDGF-BB, in murine and human fibroblast in vitro [6]. Fn14 is also upregulated in human CD14+ monocytes by INF-γ or PMA [27] and in primary BMMCs by anti-DNP IgE sensitization followed by DNP challenge [28]. However, Fn14 expression is absent in T and B lymphocytes [34].

### 2.3. Signaling Pathways Activated by TWEAK-Fn14 Interaction

When TWEAK binds to the extracellular domain of Fn14, it produces a receptor trimerization [4] (Figure 1B). This trimeric structure induces the recruitment of TRAF2 and TRAF5 though the TRAF-binding motif (PIEET) in the cytoplasmic tail, and leads the activation of different signaling pathways [14,35]. TWEAK-Fn14 binding activates several signal transduction pathways that implicate both canonical and non-canonical NF-kB pathways [14,36], mitogen-activated protein kinases pathway (MAPK) [37], PI3K/AKT [38], JAK/STAT signaling pathway [39,40], and transforming growth factor-β-activated kinase 1 [41]. MAPK activation induced by recombinant soluble TWEAK (rTWEAK) has been reported in endothelial cells [33], Thp-1 monocytic cell line [26], and cardiomyocytes [30]. In addition, rTWEAK activates PI3K/AKT in endothelial cells [38]. Although sTWEAK is responsible for the response associated with Fn14, it has been reported that mTWEAK can, in a juxtacrine manner, bind to Fn14 and activate the NF-kB signaling pathway [42].

TWEAK/Fn14 interaction triggers pathophysiological cell functions that are dependent on the cell type and the microenvironment. In this sense, it has been previously demonstrated that TWEAK participates in several pathologies that course with cardiovascular remodeling, promoting proliferation, migration, differentiation, apoptosis, inflammation, angiogenesis, and matrix degradation [43] (Summarized in Table 1). However, since TWEAK has beneficial or deleterious effects depending on the disease stage the role of TWEAK in different pathological situations precise more characterization.

In 2007, CD163 was identified as a second receptor for TWEAK [52]. CD163 is exclusively expressed by monocytes/macrophages and is a scavenger receptor for hemoglobin [53]. In addition, CD163 also acts as a scavenger receptor for TWEAK, preventing its deleterious biological effects [54]. Although CD163 interacts with TWEAK to regulate tissue regeneration after ischemic injury in vivo [55], the relevance of TWEAK/CD163 interaction in the context of cardiovascular disease needs to be explored.

## 3. TWEAK and Atherosclerosis

Atherosclerosis refers to the accumulation of fibrofatty material in the intima layer of the arteries, characterized by chronic inflammation and excessive cell proliferation [56]. It is the most common underlying pathology of coronary artery disease, peripheral artery disease, and cerebrovascular disease [57]. In the initiation of vascular lesion, low-density lipoprotein (LDL) particles accumulate in the subendothelial space of the large arteries, where they can be subject to oxidation or other modifications. These cholesterol particles activate the endothelium, and trigger the expression of adhesion molecules in their membrane, such as intracellular adhesion molecules (ICAMs), selectins, and vascular adhesion molecules (VCAMs). Circulating monocytes, T lymphocytes, and neutrophils are attracted by chemokines, binding to adhesion molecules and starting a pro-inflammatory response. Once in the intima, monocytes maturate into macrophages that express scavenger receptors, triggering the uptake of oxidized low-density lipoproteins (ox-LDL) and become foam cells. In addition, SMCs from the media migrate and proliferate into the intima in response to chemokines and cytokines secreted by inflammatory cells, forming the neointima [57,58,59]. The evolution of the atherosclerotic plaque progress by continued proliferation of SMCs and accumulation of lipid-loaded cells. In addition, extracellular matrix molecules (such as interstitial collagen and elastin, as well as proteoglycans and glycosaminoglycans) synthesized by SMCs develop the fibrous cap that confers resistance to rupture. However, continuous ingestion of ox-LDL by foam cells undergoes cell death and contributes to the necrotic core formation, which, together with the increase production of proteases (e.g., matrix metalloproteinases, MMPs) by macrophages and SMCs, promotes plaque instability. Fracture of the fibrous cap undergoes the rupture of the atheroma plaque and the occlusion of an artery by thrombus formation, causing myocardial infarction, stroke or peripheral vascular disease [57,59].

From a molecular point of view, the TWEAK-Fn14 pathway has been associated with different steps of atherosclerotic plaque development.

### 3.1. Plaque Initiation

It is well established that cumulative exposure of an artery to LDL remains a principal determinant of endothelial activation during disease initiation [46]. Interaction of rTWEAK with Fn14 can regulate the expression of different adhesion molecules, such as ICAM-1 and E-selectin in cultured human umbilical endothelial cells [46]. Moreover, rTWEAK also increases the secretion of interleukin-8 and CCL2 by endothelial cells, which predominantly recruit monocytes and neutrophils [60].

### 3.2. Lesion Progression

Plaque progression moves forward infiltration of inflammatory cells, which, together with continuous accumulation of lipids, trigger a chronic vascular inflammatory environment. Cytokines secreted by the inflammatory cells contribute to phenotypic changes in SMCs, which are transformed from a quiescent contractile phenotype into a proinflammatory phenotype, with the ability to proliferate and migrate [61]. In this sense, treatment of SMCs with rTWEAK favors the vascular phenotypic switching, increasing osteopontin and decreasing α -actin and calponin gene expression. Moreover, the number of osteopontin positive SMCs increases in atherosclerotic plaques from TWEAK/ Apolipoprotein E (ApoE) double deficient mice, confirming the role of TWEAK on SMCs phenotypic changes in vivo [39]. Both, inflammatory cells and SMCs favor plaque progression by increasing cytokines and MMPs expression. NF-kB activation plays a key role in vascular inflammation. In this regard, activated NF-kB has been shown in SMCs, macrophages, and endothelial cells of mouse and human atherosclerotic plaques [62,63]. In this context, rTWEAK injection exacerbates the inflammatory response via NF-kB activation in atheroprone mice, increasing macrophage content and plaque size [64]. In addition, genetic deletion of TWEAK or anti-TWEAK Monoclonal antibody (mAb) treatment decreases CCL2 and CCL5 protein expression in atherosclerotic plaques, and increases features of plaque stability in hyperlipidemic ApoE deficient mice [65]. Recombinant TWEAK injection also increases inflammation in a model of atherosclerosis progression under high-glucose conditions (streptozotocin-induced diabetic ApoE deficient mice) [39]. In vitro loss-of-function experiments in cultured SMCs, in vivo inhibition of TWEAK by genetic deletion, or pharmacological intervention showed that TWEAK regulates CCL5, CXCL10, and ICAM-1 mRNA expression through Signal Transducer and Activator of Transcription 1 (STAT-1) phosphorylation [39].

Although SMCs play a key role in the development of atherosclerotic plaques, macrophages are also very important players in the atherosclerotic process. Several studies have supported the role of the TWEAK/Fn14 axis on monocytes/macrophages biology during atherosclerosis. In fact, TWEAK signaling blockade by Fn14-Fc in bone marrow-derived macrophages (BMDM) decreases lipid uptake [48]. Moreover, rTWEAK induces several proinflammatory mediators of atherogenesis, such as IL-6, CCL2, and interleukin-8 (IL-8), in cultured monocytes [66]. TWEAK has been also associated with the secretion of the DNA-binding cytokine high mobility group box 1 (HMGB1) in human macrophages [38]. HMGB1 is a critical mediator of inflammation, release from necrotic cells, and activated macrophages. This DNA-binding molecule stimulates proinflammatory cytokines, chemokines, and adhesion molecules expression, triggering an inflammatory response in atherosclerotic plaques [67]. Systemic injection of rTWEAK increases the expression of HMGB1 in atherosclerotic plaques of hyperlipidemic ApoE-deficient mice. In addition, HMGB1 expression colocalizes with Fn14 expression in human atherosclerotic plaques, especially in macrophages rich-areas [38]. TWEAK/Fn14 interaction induces the release of HMGB1 via NF-kB in monocytes, which could in turn induce NF-kB activation again, forming an inflammatory positive loop [38].

Oxidative stress is also associated with inflammation and the development of atherosclerosis [68]. The increase in reactive oxygen species (ROS) production is produced in part by the activation of NADPH oxidases [69]. As a consequence, excessive ROS can trigger protease secretion, facilitating fibrous cap rupture and thrombosis [70]. In this context, in vitro experiments in macrophages have demonstrated that TWEAK and Fn14 participates in NADPH oxidase activation, regulating ROS production. Thus, genetic deletion of TWEAK in a model of atherosclerosis increases atheroma oxidative stress signals. In addition, high expression of TWEAK and Fn14 is observed in macrophage-rich areas of human atherosclerotic plaques, colocalizing with the membrane-associated subunits of the NADPH oxidase, p22phox, and the gp91phox isoform (Nox2) [71].

### 3.3. Plaque Stability

Integrity of the fibrous cap determinates the onset of the atherosclerostic plaques. Stable plaques are characterized by a thick fibrous cap, with little inflammation, macrophage accumulation, and apoptosis. In contrast, plaques with large lipid cores covered by a thin fibrous cap are more prone to rupture. The deterioration of the fibrous cap is dependent on the MMP activity of macrophages and SMCs that degrade the interstitial collagen [72]. TWEAK, Fn14, and different MMPs are co-expressed in macrophage-rich areas of human atherosclerotic plaques. Immunohistochemical staining of human atherosclerotic plaques revealed that the expression patterns of TWEAK and Fn14 in macrophages/foam cell-rich regions overlap with MMP expression [73]. In addition, Fn14 activation by rTWEAK increases MMP-1, -9, and -13 in human monocyte cell line (THP-1) [73], and systemic injection of rTWEAK is able to enhance MMP-9 and -2 protease activity in ApoE knockout mice [65]. In contrast, anti-TWEAK mAb treatment diminishes MMP activity in aortic root plaques of ApoE deficient mice [65]. The activation of MMPs is also associated with plaque calcification, a phenomenon that may compromise the stability of the plaques [74]. Pro-calcific properties of TWEAK have been described in human SMCs. TWEAK/Fn14 interaction favors human SMC osteogenic transition, decreasing α-actin and myosin heavy chain 11, and increasing bone morphogenetic protein 2, tissue non-specific alkaline phosphatase, and MMP9 activity [49]. In addition, genetic deletion of TWEAK decreases vascular calcification in atherosclerotic plaques of ApoE-deficient mice [65].

### 3.4. Plaque Rupture

The most common cause of acute thrombosis of coronary arteries is the rupture of atherosclerotic plaques. The dysfunctional endothelium of atheroma plaque loses its normal homeostatic properties, increasing adhesion molecules, chemokines, and cytokines expression, and diminishing production/availability of nitric oxide. Under these circumstances, endothelial cells produce tissue factor (TF), a potent procoagulant molecule, and plasminogen activator inhibitor 1 (PAI-1), a key endogenous inhibitor of the fibrinolysis factor. Both molecules promote a prothrombotic state and play a crucial role in vascular diseases [75]. It has been shown that Fn14 colocalizes with PAI-1 and TF in human atherosclerosis plaques [50]. In addition, rTWEAK increases PAI-1 and TF expression in cultured human SMCs. PAI-1 and TF expression is also increased in atherosclerotic plaques after rTWEAK injection in ApoE deficient mice. Conversely, treatment with anti-TWEAK antibody, anti-Fn14 antibody, or Fn14 small interfering RNA diminishes PAI-1 and TF in cultured SMCs [50].

## 4. TWEAK and Restenosis

Despite improvements in treatment of atherosclerosis by control of the risk factors, significant residual disease still remains. Percutaneous transluminal coronary angioplasty has been widely used to open up blocked coronary arteries [76]. However, many patients display restenosis or intimal hyperplasia formation after a percutaneous transluminal angioplasty. Vascular restenosis or blood vessel re-narrowing after percutaneous coronary intervention is defined as the healing response of the arterial wall to mechanical injury. It is mainly caused by an exacerbated SMC proliferation and migration combined with decreased apoptosis, which leads to a narrowing of the artery [77,78]. Long-term treatment of intimal hyperplasia postangioplasty is markedly improved by the use of drug-eluting stents, which are basically focused on the inhibition of SMCs proliferation. However, in-stent restenosis still occurs in 10% of cases [79].

Recombinant soluble TWEAK induces proliferation and migration in several cultured cell types including endothelial and aortic SMCs [33,44,60]. However, rTWEAK fails to induce proliferation in Fn14-deficient SMCs, indicating a direct effect of TWEAK/Fn14 interaction on cell proliferation [21]. rTWEAK induces the transition of the cell cycle from G0/G1 to S phase in SMCs, increasing the total number of cells in cell cycle assays. Therefore, rTWEAK increases SMCs migration in transwell and wound healing assays [21]. Mechanistically, TWEAK/Fn14 axis increases SMCs proliferation through upregulation of cyclin D1 and CDK4/6, and downregulation of Cdkn2b (p15INK4B), a highly conserved cell-cycle regulator and tumor suppressor gene. Moreover, p15INK4B and cyclin D1 expression were regulated by ERK1/2 and Akt kinases [21]. An in vivo study has also reported that Fn14 mRNA expression is upregulated in proliferating endothelial cells and vascular SMCs after 8 days of balloon injury in rats [11]. Moreover, TWEAK, Fn14 mRNA, and protein expression levels increased in the SMCs of femoral arteries after 14 days of wire injury in mice, compared with non-injured arteries [21]. In addition, TWEAK and Fn14 are expressed in the SMCs of human coronary arteries with stenosis as well as in-stent restenosis [21]. Genetic deletion of Fn14 or TWEAK, as well as pharmacological intervention with an anti-TWEAK antibody, reduces neointimal hyperplasia after wire injury in mice [21]. TWEAK-Fn14 has been also associated with the pro-apoptotic signaling in the prolonged exposure of SMCs to cleaved PAI-1, leading to SMC apoptosis [80].

## 5. TWEAK and Abdominal Aortic Aneurysm

Abdominal aortic aneurysm (AAA) is a disease that affects 5% of elderly men and is responsible for a high number of deaths in Western countries [81]. Usually AAA remains asymptomatic, and it can be identified by an incidental routine imaging. However, the sudden rupture of an AAA in affected patients is associated with high morbidity and high mortality. Moreover, no specific drug treatments have been approved for clinical use in this disease, and endovascular or open repair is currently the only therapy when the aortic diameter is >5–5.5 cm [82]. Therefore, better understanding of the pathophysiology of AAA needs to be implemented to find novel therapeutic strategies.

TWEAK and Fn14 are expressed in human AAA colocalizing with macrophages and SMCs [83] and the role of TWEAK/Fn14 axis has been demonstrated in the experimental model of elastase-induced AAA [84]. A main pathological feature of AAA includes: extracellular matrix remodeling, loss of SMCs, accumulation/activation of inflammatory cells, and formation of intraluminal thrombus [85]. Infiltration of inflammatory cells (macrophages, T cells, neutrophils, and dendritic cells) is a crucial process in AAA development, driving the progressive and pathological remodeling of the aorta [85]. There are three major chemokine/chemokine-receptor pathways controlling recruitment of circulating monocytes: CCL2/CCR2, CCL5/CCR5, and CX3CL1/CX3CR1, which are the most studied in the context of AAA [86,87]. Expression of CCL2 positively correlates with macrophage infiltration into aortic walls and acts as a promoter of AAA formation and development [88]. CCL2 and CCL5 mediate experimental aneurysm formation [89,90]. rTWEAK increases both CCL2 and CCL5 secretion in SMCs and CCL5 secretion in BMDM in a dose-dependent manner [84]. Therefore, genetic deletion of TWEAK or Fn14 diminished CCL2 and CCL5 expression, reducing monocyte and neutrophils recruitment in experimental AAA in mice [84]. Inflammatory cells and SMCs are responsible for the upregulation of MMPs during aneurysmal growth, particularly MMP-2 and MMP-9 [91]. In this sense, it is important to note that rTWEAK is also able to increase MMP-9, but not MMP-2, expression and activity in SMCs, or BMDM [65,84]. Moreover, TWEAK or Fn14 deficient mice show less MMP activity associated with MMP-9 and MMP-3 downregulation in a mouse model of elastase-induced AAA [84]. As a result, TWEAK or Fn14 deficient mice are less prone to develop AAA.

## 6. TWEAK and Heart Failure

Cardiac remodeling is the main pathophysiological basis of heart failure. This remodeling process comprises structural and functional changes, including cardiomyocyte proliferation, hypertrophy, necrosis, apoptosis, autophagy, interstitial fibrosis, contractile dysfunction, and ventricular dilatation [92]. The role of TWEAK/Fn14 axis in cardiac remodeling has been extensively studied [93]. Both, mouse and human cardiomyocytes express Fn14 [23] and its expression is upregulated by mechanical stretch or different stimuli, such as fibroblast growth factor 1, norepinephrine, and angiotensin II [93]. TWEAK and Fn14 are also markedly upregulated in cardiomyocytes after experimental myocardial infarction (MI) in vivo [30].

Cardiomyocytes proliferate during prenatal development; however, it has been described that adult cardiomyocytes proliferation is also possible during pathological ventricular remodeling [94]. Recombinant TWEAK induces rat cardiomyocytes proliferation by activation of PI3K, ERK, and GSK-3b signaling pathways [31]. In addition, rTWEAK also increases rat cardiac fibroblasts proliferation via NF-kB activation [32]. Cardiac hypertrophy in response to hemodynamic changes has two different patterns of development; eccentric and concentric hypertrophy. During eccentric hypertrophy, the heart increases in size, but only through the lengthening of myocytes, usually with loss of cell width. However, concentric hypertrophy occurs when there is a relative increase in the width of individual cardiac myocytes [95]. Elevated circulating levels of sTWEAK induced via transgenic mice, or by adenoviral-mediated gene expression in mice, result in dilated cardiomyopathy with subsequent severe cardiac dysfunction [23]. This phenotype is the consequence of cardiomyocyte elongation and cardiac fibrosis but not cardiomyocyte apoptosis [23]. In the same way, Fn14 overexpression in cultured adult rat cardiomyocytes enhances cardiomyocyte size and promotes cardiomyocyte growth. Moreover, Fn14-deficient mice exhibit less ring ventricular hypertrophy after pulmonary artery banding than wild type littermates [51].

Myocardial remodeling is characterized not only by alterations of the cardiomyocyte but also by changes in the interstitial cells and matrix [3]. rTWEAK increases cytokine synthesis and collagen expression in cardiac fibroblast by TRAF3IP2 expression, p38 MAPK, NF-kB, and AP-1 activation [96]. Furthermore, treatment of rat cardiomyocytes with rTWEAK also promotes an inflammatory response via NF-kB nuclear translocation and subsequent activation of its regulated-genes, CCL2 and CCL5 [30]. In the same way, in vivo systemic administration of recombinant TWEAK during 7 days, activates the same signaling pathways than in vitro, triggers interstitial fibrosis, increases systolic blood pressure (SBP) and produces contractile dysfunction in mice hearts [96]. Genetic depletion of TRAF3IP2 inhibits TWEAK-induced adverse cardiac effects in mice [96]. The mechanism by which TWEAK induces cardiomyocyte dysfunction could be related with proliferator-activated receptor gamma coactivator-1α (PGC1 α), a gene required for mitochondrial oxidative phosphorylation. rTWEAK decreases PGC1α expression via TNF receptor-associated factor 2 (TRAF2) and NF-kB [97]. In addition, Fn14 deletion or anti-TWEAK mAb treatment improves left ventricular function and increases PGC-1α levels after MI in mice [98].

Atrial fibrillation (AF) is an irregular heartbeat (arrhythmia) that can lead to blood clots, heart failure, stroke, or other heart-related complications. Atrial myocyte hypertrophy plays an essential role in AF. In this sense, Fn14 protein expression is increased in atrial appendages from patients with AF compared with normal subjects [99]. In addition, TWEAK protein expression is elevated in peripheral blood mononuclear cells from patients with AF. As commented above, TWEAK/Fn14 axis is a positive regulator of cardiac hypertrophy [97]. In the same way, it has been demonstrated that incubation of atrial myocytes (HL-1) with rTWEAK induces hypertrophy on these cells. TWEAK-induced myocytes hypertrophy is associated with JAK2/STAT3 pathway activation since Fn14-specific siRNA decreases JAK2/STAT3 activation and hypertrophy of atrial myocytes [99].

After an acute MI, myocyte apoptosis is a key determinant of cardiac recovery. The degree of cardiomyocytes apoptosis is vital to reduce the scar area and improve heart function [100]. Thus, pharmacological treatments to inhibit cardiomyocyte apoptosis are able to reduce the scar area and improve heart function [101,102]. In this sense, a study has described a cardioprotective role of TWEAK/Fn14 axis in models of myocardial ischemia and reperfusion. rTWEAK protects cardiomyocytes from apoptosis by activation of cardioprotective signaling PI3K/AKT [47]. In addition, rTWEAK treatment prior to reperfusion inhibits myocyte death and improves heart function and scar size in rats [47]. Overall, TWEAK studies in heart failure suggest the dual role of this cytokine depending on the time point analyzed, cell type, and environment. Nevertheless, more studies underlying the potential cardioprotection of TWEAK are needed in the future.

## 7. TWEAK and Stroke

Stroke is defined as the brain injury following transient or permanent focal cerebral ischemia, due to an occlusion of a cerebral blood vessel [103]. Stroke is the second cause of death and the first cause of disability in the industrialized world. Two types of stroke are considered; ischemic and hemorrhagic stroke, although ischemic stroke (formation of a blood clot or thrombus) accounts for the majority of cases [103]. The outcome of ischemic stroke depends on the time and space of the regional blood supply disruption and it determines the type of patient deficit. Smaller strokes may result in arm or leg weakness, and larger strokes could lead to paralysis or death. The ischemic cascade is characterized by complex pathophysiology events, as energy depletion, excitotoxicity, peri-infarct depolarization, inflammation, and apoptotic cell death. Several studies have associated TWEAK/Fn14 axis to the pathogenesis of ischemic stroke. TWEAK is widely expressed in neurons, astrocytes, and macrophages, and both TWEAK and Fn14 expression are up-regulated after ischemic strokes in humans [104], in experimental middle cerebral artery occlusion (MCAO) in mice [105,106], and in blood brain barrier (BBB) disruption in mice [107,108]. Likewise, intracerebroventricular administration of Fn14-Fc decoy receptor [106], or intraperitoneal injection of anti-TWEAK antibody, following MCAO results, in a significant reduction of infarct size by around 30–40% [105].

TWEAK is also involved in the proinflammatory response during cerebral ischemia. Treatment of cultured astrocytes with rTWEAK increase IL-6 and IL-8 secretion as well as *ICAM-1* expression [109]. In addition, rTWEAK increases CCL2 expression in astrocytes, favoring the recruitment of neutrophils [110]. The increase of CCL2 and neutrophils after MCAO was absent in TWEAK or Fn14 deficient mice, supporting the role of TWEAK in the recruitment of leukocytes into the ischemic tissue [110].

Ischemic stroke triggers disruption of the architecture of the neurovascular unit (NVU) resulting in a BBB breakdown, and contributing to the brain edema [111]. The NVU is a dynamic structure consisting of endothelial cells, basal lamina, astrocytic, pericytes, and neurons, where integrity and composition determinate the permeability of the NVU [112]. TWEAK and Fn14 expression are particularly abundant in perivascular structures, suggesting its role in the function of the NVU [113]. Intracerebral rTWEAK administration in non-ischemic wild-type mice results in NF-kB and MMP-9 activation, increasing BBB permeability. Moreover, this effect is not observed when rTWEAK is injected in Fn14 deficient mice, indicating a direct effect of TWEAK on the structure and permeability of the NVU [107]. Moreover, TWEAK inhibition by treatment with an Fn14-Fc decoy receptor, or Fn14 genetic deletion, result in a significant amelioration of the NVU permeability after cerebral ischemia [108]. Finally, oxygen-glucose deprivation is associated with neuronal apoptosis. In this sense, it has been demonstrated that under oxygen-glucose deprivation conditions, rTWEAK induce cell death via NF-KB activation and PARP-1, and caspase-3 cleavage in wild type neurons, but not in Fn14 or TWEAK deficient neurons [114].

In summary, the interaction between TWEAK and Fn14 may play an important role during cerebral ischemia, suggesting that this axis may be a new therapeutic target for acute cerebral ischemia.

## 8. Conclusions

**Figure 2:**
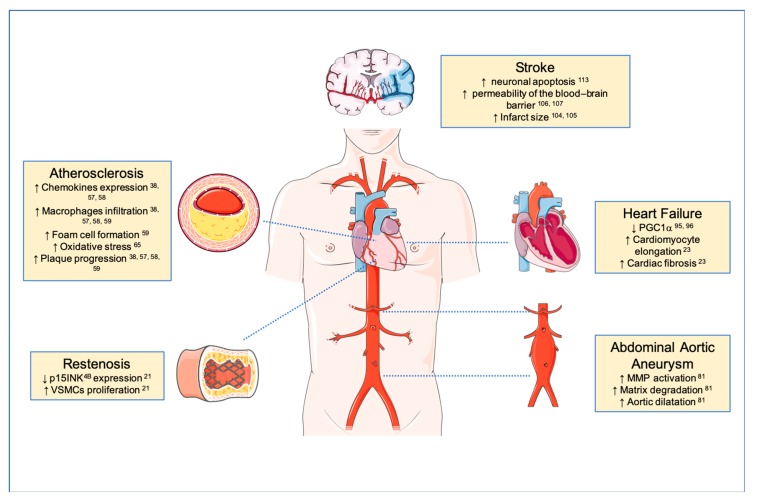
TWEAK/Fn14 axis in cardiovascular disease. Schematic representation of TWEAK roles in pathological cardiovascular remodeling diseases. TWEAK in cardiovascular diseases (CVDs) affect inflammatory cells, cardiomyocytes, fibroblasts, endothelial and smooth muscle cells (SMCs) in CVDs. TWEAK/Fn14 axis interaction promotes pathological tissue remodeling; atherosclerosis (inflammation, lipid accumulation and plaque progression, and instability), restenosis (SMCs proliferation, migration, and cyclins regulation), heart failure (cardiomyocytes dysfunction and fibrosis), abdominal aortic aneurysm (inflammation, matrix degradation, AAA progression, angiogenesis).

The studies summarized in this review highlight the key role of TWEAK/Fn14 during pathological cardiovascular remodeling (Figure 2). Data from cultured cells and different animal models make TWEAK/Fn14 axis a promising target for the treatment of cardiovascular remodeling. Several groups are developing TWEAK- or Fn14-targeted agents for possible therapeutic use in patients. These agents include monoclonal antibodies, fusion proteins, and immunotoxins [115]. Anti-TWEAK neutralizing antibody or Fn14-Fc treatment, have demonstrated a beneficial effect on atherosclerotic plaque development and restenosis post-angioplasty. Fn14 or TWEAK deletion also reduces AAA progression in mice. In addition, Fn14 deletion or anti-TWEAK treatment reduce cardiac dysfunction and the volume of the ischemic lesion after stroke. Although some of the agents developed to inhibit TWEAK/Fn14 interaction have already entered in different clinical trials, so far none of them have been used to prevent pathological vascular remodeling. For that reason, the potential use of these drugs needs to be explored in the human context.

## Figures and Tables

**Table 1:** TWEAK is a multifactorial cytokine that regulates biological processes in different cardiac and vascular cell types.

Cellular Response	Cell Type(s)	Pathology	References
Proliferation	Human endothelial cells	In vitro	[44]
	Human smooth muscle cells	In vitro	[44]
	Rat endothelial cells	Angiogenesis in rat corneas	[44]
	Mice endothelial cells	Arthritis	[45]
	Mice smooth muscle cells	Restenosis	[21]
	Post-natal rat cardiomyocytes	In vitro	[31]
	Rat cardiac fibroblasts	In vitro	[32]
Migration	Human endothelial cells	In vitro	[33,46]
	Rat aortic smooth muscle cells	In vitro	[35]
	Mice smooth muscle cells	In vitro	[21]
Survival	Cardiomyocyte cell line H9C2	In vitro/rat ischemia/reperfusion model	[47]
Differentiation	Mice Smooth muscle cells. (Contractile-Synthetic)	In vitro/In vivo in diabetes- induced atherosclerosis in ApoE^-/-^ mice	[35]
	Human endothelial cells (Endothelial activation/Expression of Adhesion molecules)	In vitro	[46]
	Mice endothelial cells (Endothelial activation/Expression of Adhesion molecules)	In vivo in diabetes induced atherosclerosis in ApoE^-/-^ mice	[35]
	Bone marrow-derived macrophages. (Lipid uptake/Foam cells formation)	In vitro	[48]
	Human vascular smooth muscle cells (Osteogenic transition/Calcification)	In vitro	[49]
	Human aortic vascular smooth muscle cells (Prothrombotic phenotype/Expression of Prothrombotic factors)	In vitro	[50]
	Adult rat cardiomyocytes (Hypertrophy)	In vitro	[51]
